# Characterization of an Atlantic cod (*Gadus morhua*) embryonic stem cell cDNA library

**DOI:** 10.1186/1756-0500-2-74

**Published:** 2009-05-06

**Authors:** Pål A Olsvik, Elisabeth Holen

**Affiliations:** 1 National Institute of Nutrition and Seafood Research, Nordnesboder 2, N-5005 Bergen, Norway

## Abstract

**Background:**

The Atlantic cod is an ecologically and economically important North Atlantic fish species and also an emerging aquaculture species. To study gene expression in Atlantic cod embryonic stem (ES) cells, our goal was to generate and analyze expressed sequence tags (ESTs) from an ES cell cDNA library of mRNA consisting of approximately 3,900 ESTs.

**Results:**

We sequenced 3,935 EST clones using a directional cDNA library made from pooled ES cells harvested at the blastula stage. Quality filtering of these ESTs allowed identification of 2,719 high-quality sequences with an average length of 442 bp containing 368 contigs and 1,276 singletons (1,644 unique sequences). BLASTX searches produced 889 significant (E-value < 10^-3^) hits, of which 698 (42.5%) were annotated with Gene Ontology terms (E-value < 10^-6^). The number of unknown unique sequences was 946 (57.5%). All the high-quality EST sequences have been deposited in GenBank (GenBank: 2,719 sequences in UniGene library dbEST id: 22,021). Gene discovery and annotations are presented and discussed.

**Conclusion:**

This set of ESTs represents one of the first attempts to describe mRNA in ES cells from a marine cold-water fish species, and provides a basis for gene expression studies of Atlantic cod ES cells.

## Background

Embryonic stem (ES) cells in culture and their development into different lineages is a unique model system that provide means to identify extracellular factors that influence embryonic cell differentiation and proliferation. ES cells are unique in their capacity to self-renew and to differentiate into multiple cell types. During differentiation, specific transcription factors activate the expression of genes that are required for each cell lineage. In addition, epigenetic regulation also appears to be a key mechanism for maintaining pluripotency and determining lineage specification [1]. ES and embryoid bodies (partly differentiated cells) can be utilized to identify and characterize factors or genes including nutrients, growth factors and hormones that may affect cell proliferation, lineage differentiation and the expression of specific genes and proteins during developmental differentiation. In fish, mRNA in oocytes and early blastocyte embryos are thought to be exclusively of maternal origin; expression of embryonic genes occurs later, at the late-blastula or gastrula stage, the exact time for this transition varies between species [2-5].

The development of gene sequence knowledge (e.g. gene annotation) is of crucial importance within the field of functional genomics. Expressed sequence tags (EST) analysis is one of the most effective means for gene discoveries, gene expression profiling, and is also one of the most efficient ways for identification of differentially expressed genes. Sequencing of ESTs from cDNA libraries specially designed from ES cells and their application to fish exposed to dietary undesirables and environmental toxicants is of importance to determine how mal-nutrition and toxicants affect cell differentiation and proliferation. EST sequencing is also the first step on the road to proteomics, a core element in functional genomics, which includes methods for detecting protein expression and for detecting protein-protein interactions. Cell-based assays designed for the detection of nutritionally or chemically induced cellular stress are of particular interest for the analysis of differentially expressed genes at different developmental stages of fish. Short-term *in vitro *assays could also be applied to study the mechanistic basis of toxicity and could offer a rapid and economically inexpensive bioassay for fish. The Atlantic cod is a key ecological species in the North Atlantic, but is also an important commercial species. In recent years many North Atlantic stocks have plummeted as a result of overfishing, triggering efforts to establish a cod aquaculture industry. Sequencing of genes, transcription factors and receptors would be of high importance to better understand how nutrition's like vitamin A and fatty acids affect cell differentiation in early life stages of fishes, contributing to the nutritional aspect of a healthy and successful cod aquaculture.

The aim of this study was therefore to generate and analyze a cDNA library for the study of gene expression in cultures of developing Atlantic cod ES cells, and to generate an EST resource for this increasingly important aquaculture species. Here we report sequencing of 3,935 EST clones, and generation of 2,719 high quality EST sequences from Atlantic cod ES cells. A brief examination of these EST sequences indicates that most of them are involved in binding (protein, DNA, RNA), catalytic (oxidoreductase), structural molecule (ribosomal) and transporter (transferase) activities.

## Methods

### 2.1. Sampling of cells

RNA harvested from ES cells was used for cDNA library construction. Newly fertilized eggs of Atlantic cod were obtained from Marin Harvest Cod, Øygarden. Eggs in the blastula stage (22–36 hour post fertilization (hpf) [2] were carefully crushed and the ES cells harvested. Cells were carefully washed and the cell pellet stored at -80°C until required. To assess changes in EST expression during differentiation by qRT-PCR cells were harvested from two separate batches of fertilized cod eggs stored at 6°C at; 22–24 hpf (day 1), 24–48 hpf (day 2) and 132–144 hpf (day 6). Newly hatched yolk-sac larvae at 1 day past hatching (dph) and larvae at 6 dph were also pooled, homogenized in Trizol and stored at -80°C for qRT-PCR analysis.

### 2.2. RNA extraction

Total RNA was extracted using phenol chloroform extraction (TRIZOL, Invitrogen, USA) and residual genomic DNA was removed by DNase treatment using DNA-*free *(Ambion, Austin TX, USA) according to the manufacturers' instructions. mRNA for construction of the cDNA library was isolated using Dynabeads mRNA Purification Kit (Dynal Biotech ASA, Oslo, Norway) according to the manufacturers' instructions. Quality and quantity of total RNA and mRNA was determined using NanoDrop ND-1000 (NanoDrop Technologies, Wilmington, DE). RNA integrity was assessed using the RNA 6000 Nano LabChip kit and Agilent 2100 Bioanalyzer (Agilent Technologies, Palo Alto, USA).

### 2.3. cDNA library construction, sequencing and data processing

A directional un-normalized library was constructed using pBluescript^® ^II XR Library Construction Kit (Stratagene Cloning Systems, La Jolla, USA) and (dT) 18 primers. The finished cDNA was inserted into the vector in a sense orientation. Plasmid vectors were ligated into *E-coli *cells. A total of 3,935 EST clones were sequenced from their 3'-ends using T3 primer (T3: 5'-AATTAACCCTCACTAAAGGGA-3'). The inserts were sequenced using the MegaBACE 1000 platform using DYEmamic ET dye terminators (GE Healthcare). All ESTs were sequenced at a commercial facility (BGI LifeTech Co. Ltd., Beijing, China) after construction of the cDNA library.

The chromatogram files obtained were processed using Phred for base calling and vector sequences from UniVec were removed using cross match [6]. Clustering, assembly and quality filtering of resulting contigs and singletons was carried out using the assembly pipeline developed at the Computational Biology Unit of BCCS at the University of Bergen. This pipeline includes repeat masking using RBR and assembly using CAP3 [7,8]. BLAST alignments of quality filtered contig and singleton sequences were then carried out against the GenBank non-redundant protein and nucleotide databases and dbEST (BLASTX and BLASTN, respectively, default parameters).

### 2.3. Sequence analysis

High-quality sequences deposited in the GenBank after base calling and vector trimming as described above were imported into the Vector NTI Advance 10 and Blast2GO softwares and further analyzed. Assembly and clustering were done using Vector NTI with default settings to obtain contigs and singletons. Gene ontology (GO) annotations were assigned using Blast2GO [9]. All 1,644 unique sequences were compared to the GenBank database as of February 2009 using BLASTX. The cut-off for sequence similarity was E-value < 10^-3 ^for the BLAST searches and <10^-6 ^for the annotation step. From these annotations, pie charts of sequence distribution were made using a 2^nd ^level analysis and filter cut-off of 30 sequences based on biological process, molecular function, and cellular component.

### 2.4. Real-time qRT-PCR

Four genes belonging to the GO "Embryonic development ending in birth or egg hatching" (GO:0009792) were selected for qRT-PCR analysis. These genes were BMI1 polycomb ring finger oncogene [GenBank:EX738556], One-eyed pinhead protein [GenBank:EX737753], Ovary-expressed homeobox protein [GenBank:EX736873] and PRP19 [GenBank: EX738721]. Two potential reference genes, GAPDH and β-actin, were analyzed for stability with the *geNorm *and *NormFinder *tools [10,11]. The PCR primers used for amplification as well as gene function are given in Table 1. Real-time quantitative RT-PCR was performed according to Olsvik et al. [12]. Briefly, a two-step real-time qRT-PCR protocol was used to quantify the mRNA levels of the genes in Atlantic cod cells and larvae. TaqMan Reverse Transcription Reagent containing Multiscribe Reverse Transcriptase (50 U/μl) (Applied Biosystems, Foster City, CA, USA) was used to make cDNA. Total RNA input was 500 ng in each reaction for all genes. No template controls (ntc) and RT-controls were run for quality assessment. Reverse transcription was performed at 48°C for 60 min by using oligo dT primers (2.5 μM) for all genes in 30 μl total volume. The final concentration of the other chemicals in each RT reaction was: MgCl_2 _(5.5 mM), dNTP (500 mM of each), 10× TaqMan RT buffer (1×), RNase inhibitor (0.4 U/μl) and Multiscribe reverse transcriptase (1.67 U/μl). 2.0 μl cDNA from each RT reaction for all genes was transferred to a new 96-well reaction plate and the real-time PCR run in 20 μl reactions on the LightCycler^® ^480 Real-Time PCR System (Roche Applied Sciences, Basel, Switzerland). Real-time PCR was performed using SYBR Green Master Mix (LightCycler 480 SYBR Green master mix (Roche Applied Sciences, Basel, Switzerland) and gene specific primers (500 nM). PCR was achieved with initial denaturation and enzyme activation for 5 min at 95°C, followed by 40 cycles of 10 s denaturation at 95°C, 20 s annealing at 60°C and 30 s elongation at 72°C. Mean normalized expression (MNE) of the four target genes was calculated according to the *geNorm *manual [10], in which the raw Ct values are transformed to quantities with PCR efficiency correction. β-actin was used as a reference gene in the final calculations since GAPDH was too unstable.

**Table 1:** PCR primers, amplicon sizes and function of genes selected for qRT-PCR analysis.

**Gene**	**Accession no.**	**Forward primer (5' – 3')**	**Reverse primer (5' – 3')**	**Size (bp)**	**Function (from mammalian homologs)**
BMI1 polycomb ring finger oncogene	EX738556	CAGGCGGCAAACAAGAGGTA	ATGACTTCAACCTGGTAGGTGTTG	113	Component of the Polycomb group (PcG) multiprotein PRC1 complex, a complex required to maintain the transcriptionally repressive state of many genes, including Hox genes, throughout development. PcG PRC1 complex acts via chromatin remodeling and modification of histones; it mediates monoubiquitination of histone H2A 'Lys-119', rendering chromatin heritably changed in its expressibility. In the PRC1 complex, it is required to stimulate the E3 ubiquitin-protein ligase activity of RNF2/RING2
One-eyed pinhead protein	EX737753	AGAACGGAGGGACGTGCAT	TCTGAACCCACTCCCCATGA	126	Involved in the correct establishment of the left-right axis. May play a role in mesoderm and/or neural patterning during gastrulation
Ovary-expressed homeobox protein (shows similarity to Nanog)	EX736873	AGCCCAATCGGGACTCACTT	TTCTTGGCATTGTAGTGCTCAGA	117	Transcription regulator involved in inner cell mass and embryonic stem (ES) cells proliferation and self-renewal. Imposes pluripotency on ES cells and prevents their differentiation towards extraembryonic endoderm and trophectoderm lineages. Blocks bone morphogenetic protein-induced mesoderm differentiation of ES cells by physically interacting with SMAD1 and interfering with the recruitment of coactivators to the active SMAD transcriptional complexes (by similarity). Acts as a transcriptional activator or repressor (by similarity). Binds optimally to the DNA consensus sequence 5'-[CG] [GA] [CG]C [GC]ATTAN [GC]-3' (by similarity). When overexpressed, promotes cells to enter into S phase and proliferation (by similarity)
Pre-mRNA-processing factor 19 (PRP19)	EX738721	GGACCGACCCGATGAATG	CCTGGAGGGACTTGAGAATGG	126	Plays a role in DNA double-strand break (DSB) repair and pre-mRNA splicing reaction. Binds double-stranded DNA in a sequence-nonspecific manner. Acts as a structural component of the nuclear framework. May also serve as a support for spliceosome binding and activity. Essential for spliceosome assembly in a oligomerization-dependent manner and might also be important for spliceosome stability. May have E3 ubiquitin ligase activity. The PSO4 complex is required in the DNA interstrand cross-links (ICLs) repair process. Overexpression of PRPF19 might extend the cellular life span by increasing the resistance to stress or by improving the DNA repair capacity of the cells

The descriptions of gene function were obtained from the GeneCards database [13] of human homologs.

## Results and discussion

### cDNA library overview

A total of 3,935 ESTs were sequenced from the cDNA library made from Atlantic cod ES cells, summarized in Table 2. After quality filtering 2,719 high-quality EST clones were identified, with an average length of 442 bp. Thus, about 30% of the sequences were filtered out; a relatively large amount of the total number of clones sequenced. 8% of the 2,719 high-quality sequences were shorter than 300 bp. The quality of the cDNA library was good, with a titer of 10^6 ^cfu/ml and a net average insert size for all clones of 1.3 bp. Cluster analysis assembled the 2,719 high-quality ESTs into 368 contigs and 1,276 singletons (1,644 unique sequences). 42.5% (698) of the 1,644 unique sequences were annotated to known genes using BLASTX with an e-value cut-off of 10^-3 ^and an annotation cut-off of 10^-6 ^with the Blast2GO software. There were 946 unknown unique sequences (57.5%) in the cDNA library. All high-quality EST sequences were deposited in the GenBank at NCBI, searchable as "*Gadus morhua *stem cell library ZNS". Accession numbers are between EX736070 and EX743943 (2,719 ESTs out of a larger number of Atlantic cod EST sequences). The 2,719 sequences from this library have also been assembled in the UniGene database [13]. 1,952 ESTs from this library were grouped into 1,001 UniGene entries (putative genes) (UniGene build #7, 22-Aug-2008).

**Table 2:** A summary of the EST analysis.

**Description**	**Number**	**Percentage**
Total number of sequences	3935	
Number of high-quality sequences	2719	69.1
Number of contigs	368	
Number of clones included in the contigs	1443	
Number of singletons	1276	
Number of unique sequences	1644	
Known unique sequences	698	42.5^1^
No BLASTX hits	755	
No annotations	101	
No mapping	90	
Unknown unique sequences	946	57.5^1^

Analyzed using Blast2GO software with a BLASTX cut-off of 10^-3 ^and a GO annotation cut-off of 10^-6^. ^1^Percentage of high-quality sequences.

### Annotations

The 22 most abundant genes from the cDNA library are listed in Table 3. These contigs consisted of 11 or more ESTs. By comparing these contigs with sequences in other Atlantic cod libraries [14] it is evident that most of these genes are not uniquely expressed in ES cells. Most abundant was H3 histone, subunit 3B. There were also several other histone genes among the most abundant genes, i.e. histone H2A family member ZA, histone H2A family member X and H3 histone, subunit 3A, as well as genes involved in transcriptional regulation of chromatin assembly such as chromobox protein homolog 3, maintenance of chromatin architecture high-mobility group box 2 and THAP-domain containing 9. Histones are the core components of the nucleosome. The nucleosome is a histone octamer containing two molecules each of H2A, H2B, H3 and H4 assembled in one H3–H4 heterotetramer and two H2A-H2B heterodimers. Nucleosomes wrap and compact DNA into chromatin, limiting DNA accessibility to the cellular machineries that require DNA as a template. Histones play a central role in transcription regulation, DNA repair, DNA replication and chromosomal stability [15]. Transcription regulation, epigenetic modifications and chromatin structures are the key modulators in controlling the pluripotency nature of ES cells [16]. Thus, histone modifications appear to be of great importance for ES cells to maintain their pluripotency, explaining the high abundance of chromatin-related transcripts in the examined ES cell cDNA library. Also among the most abundant transcripts were genes encoding proteins involved in cellular respiration including COX1, COX2 and cytochrome b as well as a number of ribosomal genes. Using the UniGene approach, the FXYD domain containing ion transport regulator 9b ranked top as the most abundant transcript (see the link above). FXYD genes encode membrane-bound regulatory proteins associated with Na, K-ATPase and thus osmoregulation adaptation in teleost fishes [17].

**Table 3:** The most abundant ESTs detected from the 2719 high-quality clones in the cDNA library.

**Cluster**	**# of sequences**	**Putative identity**	**% of total**
41	40	H3 histone, subunit 3B	1.47
170	28	Cold-inducible RNA binding protein	1.03
292	27	Histone H2A family member ZA	0.99
276	25	Cytochrome c oxidase subunit II	0.92
62	21	28S ribosomal RNA gene	0.77
79	19	High-mobility group box 2	0.70
13	17	Cytochrome b	0.63
131	17	Chromobox protein homolog 3	0.63
109	16	Unknown	0.59
229	15	Histone H2A family member X	0.55
367	12	Unknown	0.44
215	12	THAP-domain containing protein 9	0.44
142	12	Lipocalin precursor	0.44
64	12	Cytochrome c oxidase subunit I	0.44
302	12	Unknown	0.44
331	12	Small nuclear ribonucleoprotein polypeptide D1	0.44
159	12	Ribonucleoside-diphosphate reductase subunit M2	0.44
36	12	Small nuclear ribonucleoprotein polypeptide D3	0.44
77	12	H3 histone, subunit 3A	0.44
182	11	18S ribosomal RNA gene	0.40
201	11	18S ribosomal RNA gene	0.40
140	11	Unknown	0.40

Assembly and clustering were done with default settings in ContigExpress (a component of the Vector NTI Advance 10.3.0. software).

### Gene ontology

**Figure 1:**
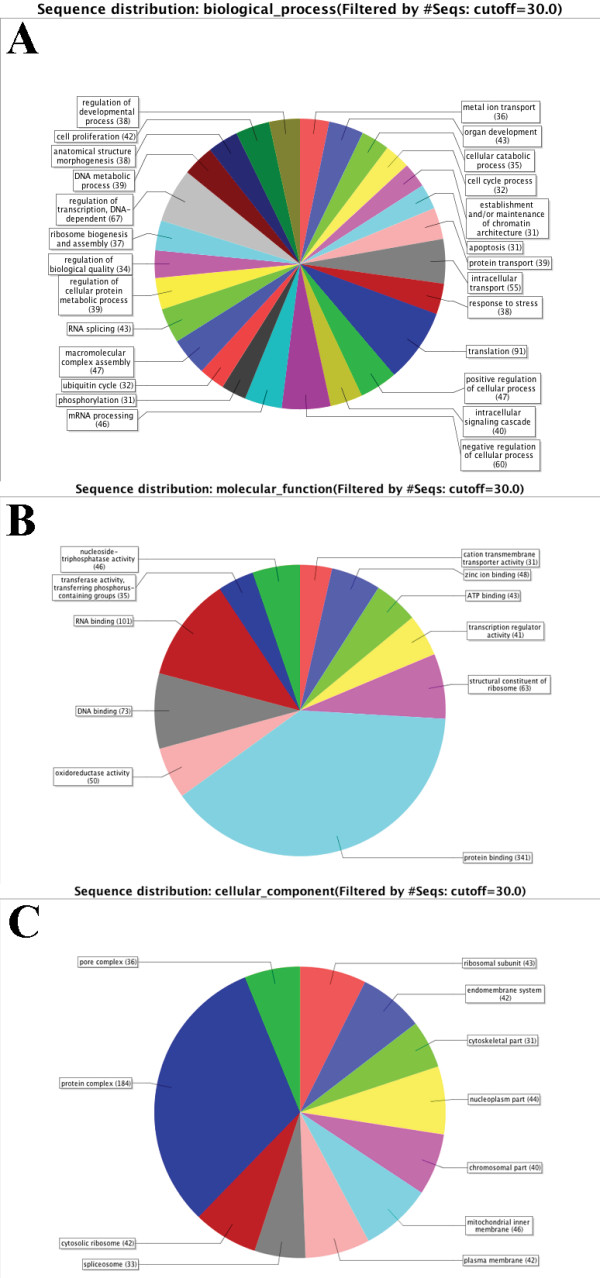
**Gene ontology (GO) graphs using percentages of 2^nd ^level GO terms**. Overall, 696 high-quality sequences were annotated using Blast2GO software and included in the graphs. Each of the three categories is presented, including biological process (A), molecular function (B) and cellular component (C). The number of GOs in each class is shown, restricted to GO classes with 30 or more entities.

The high-quality ESTs were compared to annotations through the Gene Ontology Consortium [18] using Blast2GO. Filtered with 30 sequences cut-off, the 696 known unique sequences with GO hits were used to create graphs based on percentages of 2^nd ^level GO terms (Fig. 1). The five most common biological processes were translation, regulation of transcription, negative or positive regulation of cellular processes and intracellular transport (Fig. 1A). Comparing all 2719 sequences, including redundant ESTs, the most common biological processes also included nucleosome assembly and regulation of developmental processes. In the category of molecular function, the five most abundant transcripts were involved in protein, DNA and RNA binding as well as oxidoreductase and structural constituent of ribosome activities (Fig. 1B). Under the cellular component category, most transcripts were linked to the protein complex, as well as to membranes, ribosomes and the nucleosome (Fig. 1C). Although the most abundant GO terms are linked to rather common ontologies, the high ranking of GO terms like nucleosome assembly and regulation of developmental processes clearly demonstrate the uniqueness of the library. In order to further evaluate the uniqueness of the sequences from the ES cell library, the annotations of the 2,719 ESTs in this library were compared to two other cDNA libraries obtained from early developmental stages in Atlantic cod; *G. morhua *embryo library (UniGene library dbEST id: 23,116 containing 10,821 ESTs from embryos at the cleavage stage) and *G. morhua *larvae library (dbEST id: 23,099 containing 17,519 ESTs). Comparing the top-ranking ESTs from these three libraries using Digital Differential Display (DDD) and Fisher Exact Test, the UniGene tools developed to compare EST profiles in order to identify genes with significantly different expression levels, it is evident that chromatin-related transcripts involved in restriction of transcription are especially abundant in the examined ES cell library.

### qRT-PCR data

**Figure 2:**
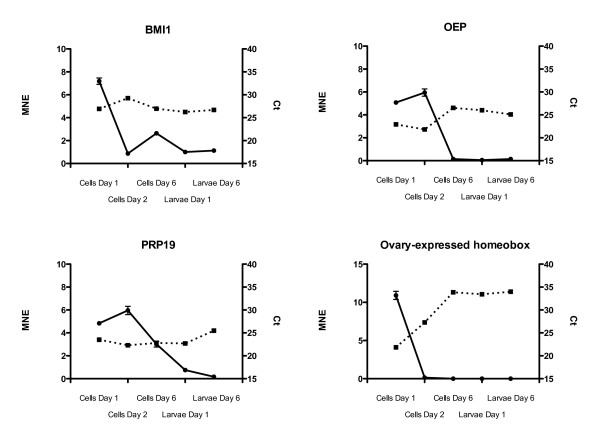
**Transcriptional levels of BMI1 polycomb ring finger oncogene (BMI1), One-eyed pinhead protein (OEP), Pre-mRNA processing factor 19 (Prp19) and Ovary-expressed homeobox protein in Atlantic cod embryos at 1, 2 and 6 dpf (n = 3), as well as in larvae at day 1 and 6 (n = 1)**. At the blastula and gastrula stages, cells from about 100 eggs were used; yielding approximately 100,000 cells in cells at day 2 and many more cells at day 6. 10 larvae were pooled together for each of the larvae day 1 and day 6 samples. Mean normalized expression (MNE) – whole line. Raw Ct – dotted line.

**Figure 3:**
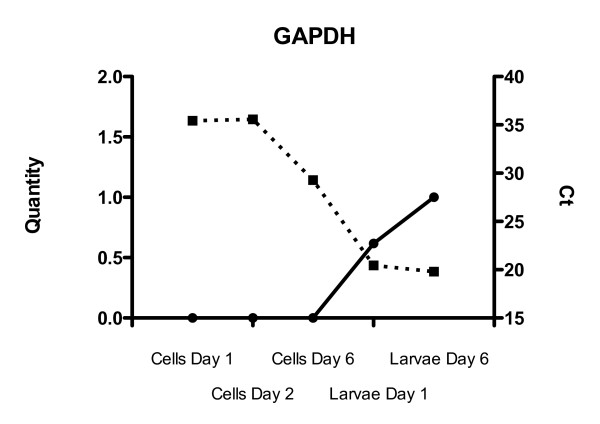
**GAPDH quantities and raw Ct values in Atlantic cod embryos at 1, 2 and 6 dpf, as well as in larvae at day 1 and 6**. Quantities – whole line. Raw Ct – dotted line.

Four randomly picked genes belonging to the GO "Embryonic development ending in birth or egg hatching" (GO:0009792) involved in maintenance of pluripotency and cell proliferation were analyzed in cells at day 1, 2 and 6 as well as in larvae at day 1 (yolk sack) and 6 in order to validate the cDNA library (Fig. 2). In many vertebrates, including teleost fish, early development of the embryo is directed by maternal transcripts and characterized by limited zygotic gene activity [4]. Genes annotated to the selected GO should therefore show clear time-dependent alterations in transcriptional levels as the embryo goes through the blastula stage and early differentiation. Based on previous qRT-PCR studies of transcripts in ES cells in mammalian systems, GAPDH and β-actin were chosen as reference genes [19]. Analyzed with *geNorm *and *NormFinder*, β-actin was relatively stably expressed and was used for data normalization (*NormFinder *variability score 0.13 ± 0.1). Raw Ct values are included in the figures for comparison of expression at time points (dotted lines, Fig. 2). One of the examined genes, ovary-expressed homeobox protein, which resembles the mammalian Nanog gene, exhibits a transcription pattern typical for ES cell markers with a characteristic post-blastula transcriptional drop. In addition, the detection of another ES cell specific gene, an ortholog of the mammalian Pou5f1, validated the authenticity of the blastula-stage cDNA library. Two transcripts, annotated to this ES cell specific marker, were found in the cDNA library. These proteins are essential for self-renewal and repression of genes initiating differentiation thus maintaining ES cell pluripotency [1,20,21]. The qRT-PCR data also suggest that zygotic transcription in Atlantic cod embryos is initiated between days 2 and 6 post fertilization. Almost no GAPDH transcription occurred in the cells at day 1 and 2 (Ct values around 35) (Fig. 3), whereas the transcription was more than 60-fold higher at day 6 (Ct around 29), suggesting increasing carbohydrate metabolism and glycolytic activity in the cells at the gastrula stage. For GAPDH, only raw Ct values and un-normalized quantities are presented (Fig. 3). Raw CT values were transformed to quantities using the comparative delta CT method where the highest relative quantity for each gene is set to 1 as required for input into *geNorm*.

## Competing interests

The authors declare that they have no competing interests.

## Authors' contributions

EH was responsible for cell sampling and all ES cell work and critically revised the manuscript. PAO analyzed the ES cell cDNA library and the qRT-PCR data and wrote the manuscript. Both authors read and approved the final manuscript.
